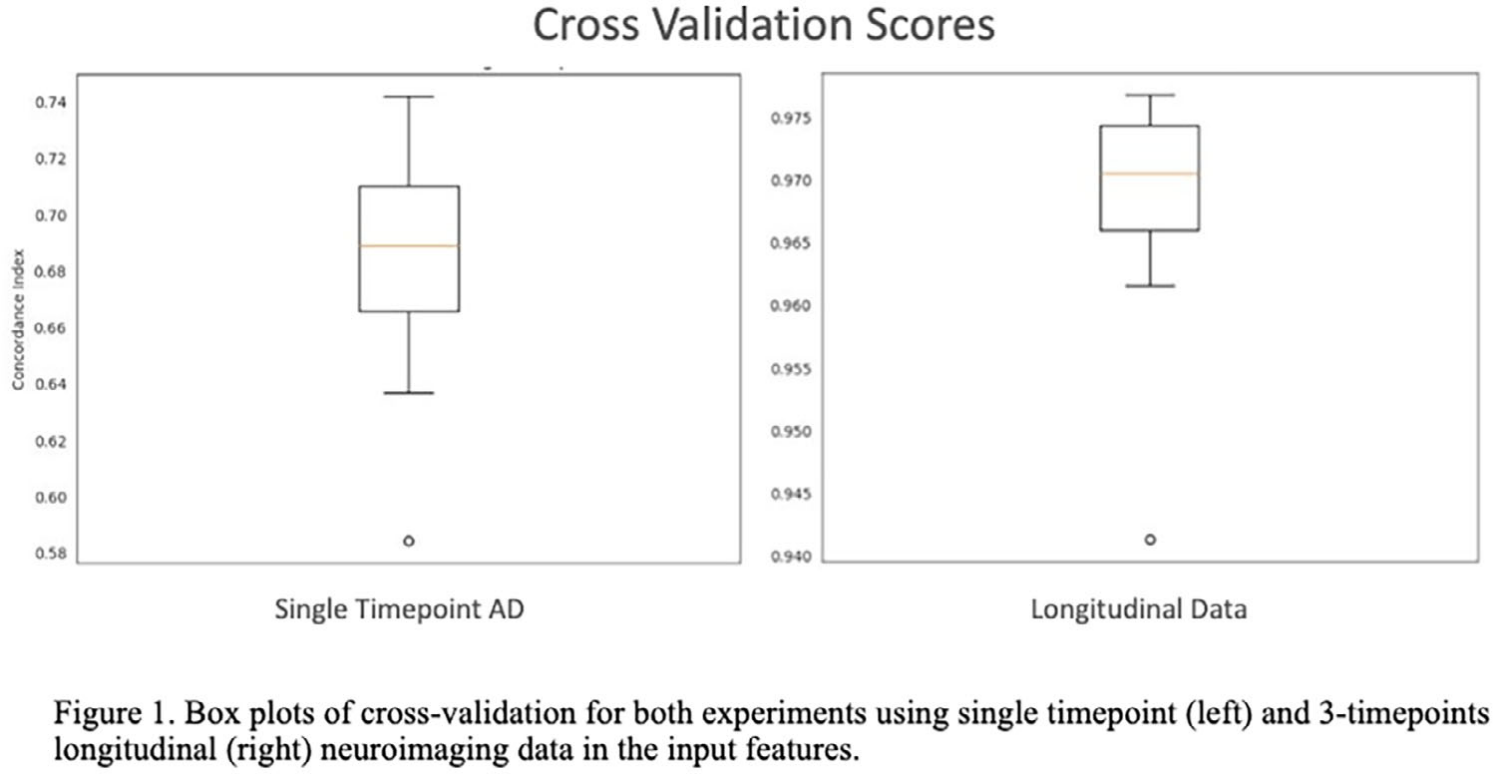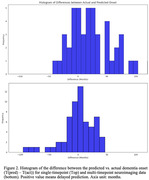# Deep Survival Analysis for Alzheimer’s Disease Based on Longitudinal Neuroimaging Data

**DOI:** 10.1002/alz.092003

**Published:** 2025-01-09

**Authors:** Da Ma, Sophia Kyriacou

**Affiliations:** ^1^ Wake Forest University School of Medicine, Winston‐Salem, NC USA; ^2^ Georgia Institute of Technology, Atlanta, GA USA

## Abstract

**Background:**

Alzheimer’s Disease (AD) accounts for more than half of dementia cases and is a leading cause of death in the United States among elderly population. Early risk prediction is crucial to ensure effective and timely intervention plans. Deep learning‐based survival analysis based on cross‐sectional neuroimaging data has shown promising results in predicting the future progression of the dementia onset at an early stage. This study aims to derive deep survival analysis using multi‐timepoint longitudinal neuroimaging data to achieve a more accurate dementia onset prediction.

**Method:**

Data: Longitudinal MRI of 1724 subjects (average age was 73.8) from the Alzheimer’s Disease Neuroimaging Initiative was used. To derive dementia onset event and duration information for the survival analysis, censored data (i.e. dementia conversion happened before or after the study period) was excluded (19.6% left‐censored, 63.2% right‐censored), resulting in 297 subjects. The input features included 89 FreeSurfer‐parcellated brain structural volumes, and demographic factors such as age and sex. Deep survival analysis was conducted using DeepSurv Pytorch library, using a multi‐layer perceptron (MLP) model architecture as the feature extractor. Image features from three timepoints were concatenated before feeding into the deep survival model for predicting future AD conversion. For comparison, ablation studies were conducted to include single timepoint neuroimaging features for each subject (derived from the last timepoints from the longitudinal features). The survival models’ performance was evaluated using the concordance index (C‐index) using 10‐fold cross‐validation.

**Result:**

The accuracy of the three‐timepoint longitudinal data (C‐index=97.1%) is higher than the single timepoint study (C‐index=75.6%) (Figure 1). Figure 2 shows the histogram of the difference between the actual and predicted dementia onset time (derived by taking the 0.5 threshold cutoff point from the C‐index, Positive value means delayed prediction). Single timepoint prediction showed more false negatives (delayed prediction) while longitudinal prediction showed relatively even distribution of false positive (earlier prediction) and false negative.

**Conclusion:**

In this study, we demonstrated the of longitudinal neuroimaging data in deep survival analysis to improve the prediction the time of conversion for future dementia, with less false negative, promoting early prediction and potential early intervention prior to the dementia.